# Supplementary material for: Causal Relationship between Aging and Anorexia Nervosa: A White-Matter-Microstructure-Mediated Mendelian Randomization Analysis
Source: Biomedicines. 2024 Aug 16;12(8):1874. doi: 10.3390/biomedicines12081874 (PMC11351342; doi:10.3390/biomedicines12081874)
Supplement: Supplementary file 1 [file biomedicines-12-01874-s001.zip › Supplementary Figure/Figure S4/Singe SNP plot of MR analysis for TL to ubm-b-1912.pdf]

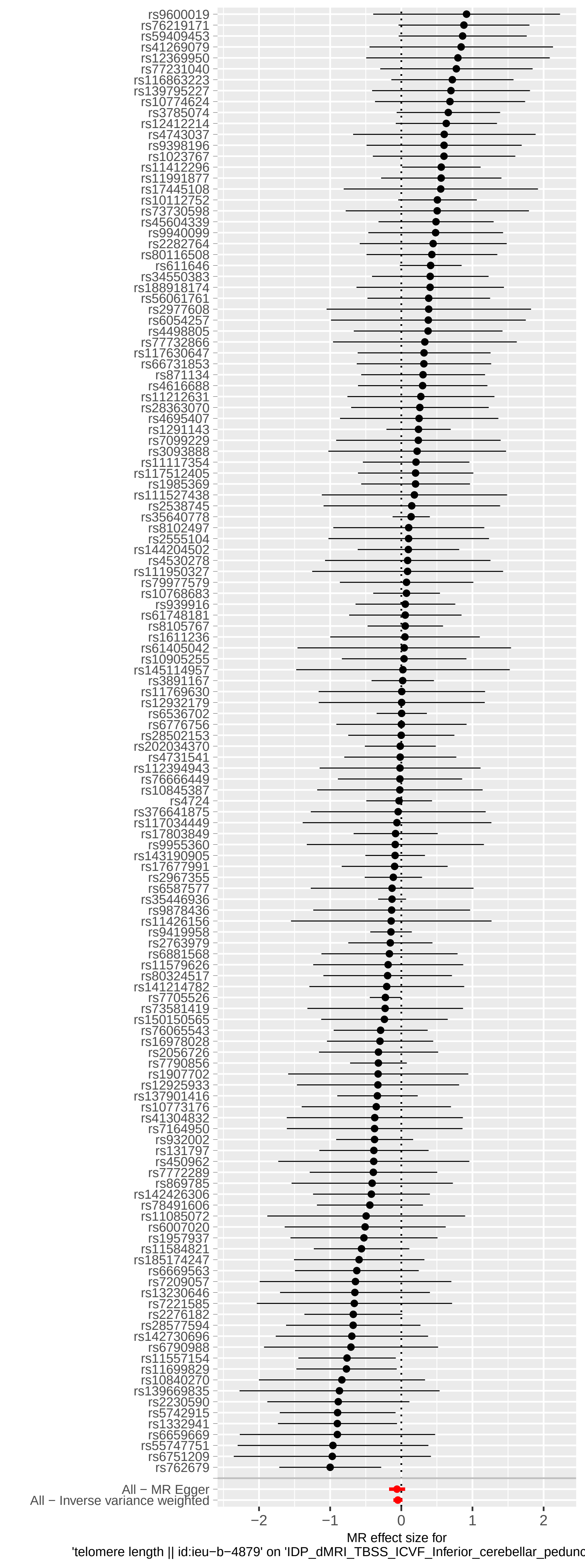
rs9600019
rs76219171
rs59409453
rs41269079
rs12369950
rs77231040
rs116863223
rs139795227
rs10774624
rs3785074
rs12412214
rs4743037
rs9398196
rs1023767
rs11412296
rs11991877
rs17445108
rs10112752
rs73730598
rs45604339
rs9940099
rs2282764
rs80116508
rs611646
rs34550383
rs188918174
rs56061761
rs2977608
rs6054257
rs4498805
rs77732866
rs117630647
rs66731853
rs871134
rs4616688
rs11212631
rs28363070
rs4695407
rs1291143
rs7099229
rs3093888
rs11117354
rs117512405
rs1985369
rs111527438
rs2538745
rs35640778
rs8102497
rs2555104
rs144204502
rs4530278
rs111950327
rs79977579
rs10768683
rs939916
rs61748181
rs8105767
rs1611236
rs61405042
rs10905255
rs145114957
rs3891167
rs11769630
rs12932179
rs6536702
rs6776756
rs28502153
rs202034370
rs4731541
rs112394943
rs76666449
rs10845387
rs4724
rs376641875
rs117034449
rs17803849
rs9955360
rs143190905
rs17677991
rs2967355
rs6587577
rs35446936
rs9878436
rs11426156
rs9419958
rs2763979
rs6881568
rs11579626
rs80324517
rs141214782
rs7705526
rs73581419
rs150150565
rs76065543
rs16978028
rs2056726
rs7790856
rs1907702
rs12925933
rs137901416
rs10773176
rs41304832
rs7164950
rs932002
rs131797
rs450962
rs7772289
rs869785
rs142426306
rs78491606
rs11085072
rs6007020
rs1957937
rs11584821
rs185174247
rs6669563
rs7209057
rs13230646
rs7221585
rs2276182
rs28577594
rs142730696
rs6790988
rs11557154
rs11699829
rs10840270
rs139669835
rs2230590
rs5742915
rs1332941
rs6659669
rs55747751
rs6751209
rs762679
All − MR Egger
All − Inverse variance weighted
−2
−1
0
1
2
MR effect size for
'telomere length || id:ieu−b−4879' on 'IDP_dMRI_TBSS_ICVF_Inferior_cerebellar_peduncl
